# Enhancing CO_2_/N_2_ and CO_2_/CH_4_ Separation Properties of PES/SAPO-34 Membranes Using Choline Chloride-Based Deep Eutectic Solvents as Additives

**DOI:** 10.3390/membranes14110230

**Published:** 2024-11-05

**Authors:** Jonathan S. Cardoso, Zhi Lin, Paulo Brito, Licínio M. Gando-Ferreira

**Affiliations:** 1 Chemical Engineering and Renewable Resources for Sustainability (CERES), Department of Chemical Engineering, Faculty of Sciences and Technology, University of Coimbra, Pólo II, Rua Sílvio Lima, 3030-790 Coimbra, Portugal; 2 Centre for Research in Ceramics and Composite Materials (CICECO), Department of Chemistry, University of Aveiro, 3810-193 Aveiro, Portugal; zlin@ua.pt; 3 Mountain Research Centre (CIMO), Polytechnic Institute of Bragança, Campus de Santa Apolónia, 5300-253 Bragança, Portugal; paulo@ipb.pt; 4 Associate Laboratory for Sustainability and Technology in Mountains Regions (SusTEC), Polytechnic Institute of Bragança, Campus de Santa Apolónia, 5300-253 Bragança, Portugal

**Keywords:** mixed-matrix membranes, PES, SAPO-34, deep eutectic solvents, interfacial voids

## Abstract

CO_2_ separation is an important environmental method mainly used in reducing CO_2_ emissions to mitigate anthropogenic climate change. The use of mixed-matrix membranes (MMMs) arrives as a possible answer, combining the high selectivity of inorganic membranes with high permeability of organic membranes. However, the combination of these materials is challenging due to their opposing nature, leading to poor interactions between polymeric matrix and inorganic fillers. Many additives have been tested to reduce interfacial voids, some of which showed potential in dealing with compatibility problems, but most of them lack further studies and optimization. Deep eutectic solvents (DESs) have emerged as IL substitutes since they are cheaper and environmentally friendly. Choline chloride-based deep eutectic solvents were studied as additives in polyethersulfone (PES)/SAPO-34 membranes to improve CO_2_ permeability and CO_2_/N_2_ and CO_2_/CH_4_ selectivity. SAPO-34 crystals of 150 nm with a high surface area and microporosity were synthesized using dry-gel methodology. The PES/SAPO-34 membranes were optimized following previous work and used in a defined composition, using 5 or 10 *w*/*w*% of DES during membrane preparation. All MMMs were characterized by their ideal gas permeability using N_2_ and CO_2_ pure gasses. Selected membranes were also tested using CH_4_ pure gas. The results presented that 5 *w*/*w*%, in polymer mass, of ChCl–glycerol presented the best result over the synthesized membranes. An increase of 200% in CO_2_ permeability maintains the CO_2_/N_2_ selectivity for the non-modified PES/SAPO-34 membrane. A CO_2_/CH_4_ selectivity of 89.7 was obtained in PES/SAPO-34/ChCl-glycerol membranes containing 5 *w*/*w*% of this DES, which is an outstanding ideal separation performance for MMMs when compared to other results in the literature. FTIR analysis reiterates the presence of glycerol in the membranes prepared. Dynamic Mechanical Thermal Analysis (DMTA) shows that the addition of 5 *w*/*w*% of DES does not impact the membrane flexibility or polymer structure. However, in concentrations higher than 10 *w*/*w*%, the inclusion of DES could lead to high membrane rigidification without impacting the overall thermal resistance. SEM analysis of DES-enhanced membranes presented asymmetric final membranes and reaffirmed the results obtained in DMTA about rigidified structures and lower zeolite–polymer interaction with higher concentrations of DES.

## 1. Introduction

Emerging membrane technologies offer promising advantages for the separation of CO_2_ from natural gas (CH_4_) and air (N_2_), including low energy consumption, ease of maintenance, straightforward operational control, low capital costs, and high energy efficiency. However, the broader adoption of polymeric membranes is limited by the inherent trade-off between selectivity and permeability, as depicted by Robeson’s upper bound curve. In contrast, inorganic membranes exceed the upper bound in performance but are challenging to process. Consequently, the performance of mixed-matrix membranes (MMMs) depends on the selective properties of inorganic fillers, while the mechanical stability and ease of production provided by polymers enhance processing [1,2,3,4].

Numerous MMMs have been synthesized using various inorganic fillers, such as silicates, zeolites, carbon nanotubes, and metal–organic frameworks (MOFs), which are usually selected according to the separation intended to be performed [5,6,7]. In this regard, SAPO-34 was selected due to its selective properties and pore size (0.38 nm) near the kinetic diameter of CO_2_ (0.33 nm), N_2_ (0.364 nm), and CH_4_ (0.38 nm), which are targets in this separation [8,9]. However, the presence of unselective voids caused by the incompatibility between organic matrix and inorganic fillers affects the separation performance of those MMMs. Attempts to enhance the compatibility led to the introduction of interactive functional groups on fillers [10,11,12].

Amine groups [13,14], silane groups [15,16], and ionic liquids [17,18] are the most used surface particle modifiers. Modifying the filler surface to enhance compatibility with the polymer is intended by adding functional groups or coatings to minimize chemical reactions or increase compatibility; several studies have attempted to use different surface modifiers to improve CO_2_/CH_4_ and CO_2_/N_2_ separation [19,20,21,22,23,24]. However, the emerging capabilities of deep eutectic solvents (DESs) arrive as a cheap and eco-friendly alternative to ionic liquids [25,26,27,28,29]. Ionic liquids (ILs) are salts that exist in a liquid state at relatively low temperatures composed of a combination of organic cations and organic or inorganic anions, presenting low volatility, high thermal stability, and conductivity [30,31]. Deep eutectic solvents (DESs) are a type of system that forms a liquid phase through a eutectic mixture of compounds. It is a combination of two or more components, typically a hydrogen bond donor (HBD) and a hydrogen bond acceptor (HBA), resulting in a substance with a melting point significantly lower than that of any of the individual component, strong intermolecular interaction between the components, biodegradability, non-toxicity, and thermal stability [32,33,34,35].

This work aimed to study the influence of choline chloride-based deep eutectic solvents in 5 *w*/*w*% and 10 *w*/*w*% concentrations, based on polymer mass, in a fixed optimized composition of a PES/SAPO-34 mixed-matrix membrane. The novelty of this study relies on how DESs can influence the final properties of MMM and if DESs are a real alternative to ionic liquids and other additives for mixed-matrix membranes. Ideal CO_2_ and N_2_ permeabilities were studied, and CH_4_ in selected cases. Membranes incorporating less than 5 *w*/*w*% do not present expressive variation in CO_2_/N_2_ selectivity or CO_2_ and N_2_ permeability. Moreover, membranes containing 20 *w*/*w*% of DES were fabricated, but they did not meet the necessary criteria for further testing. The final membranes exhibited excessive porosity or degradation, attributed to the effects of DES inclusion. Changes in structure and chemical and thermal properties caused by the inclusion of DES during the membrane fabrication were analyzed, which is vital for explaining how DESs can interfere with the membrane characteristics and separation potential.

## 2. Results and Discussion

### 2.1. Characterization of PES/SAPO-34 Mixed-Matrix Membranes

**Figure 1:**
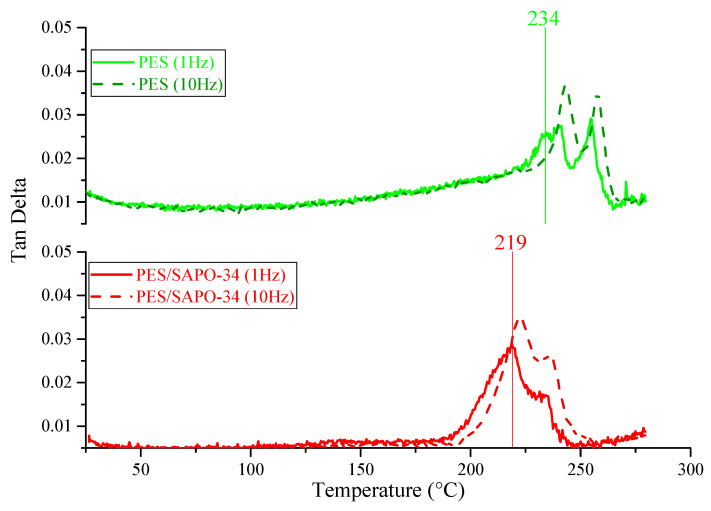
DMTA for PES and PES/SAPO-34 at 1 Hz and 10 Hz.

**Figure 2:**
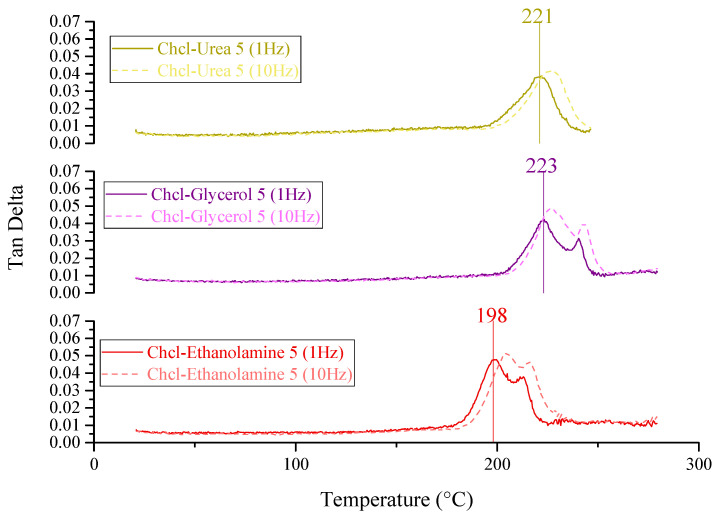
DMTA for PES/SAPO-34/DES using 5 *w*/*w*% at 1 Hz and 10 Hz.

**Figure 3:**
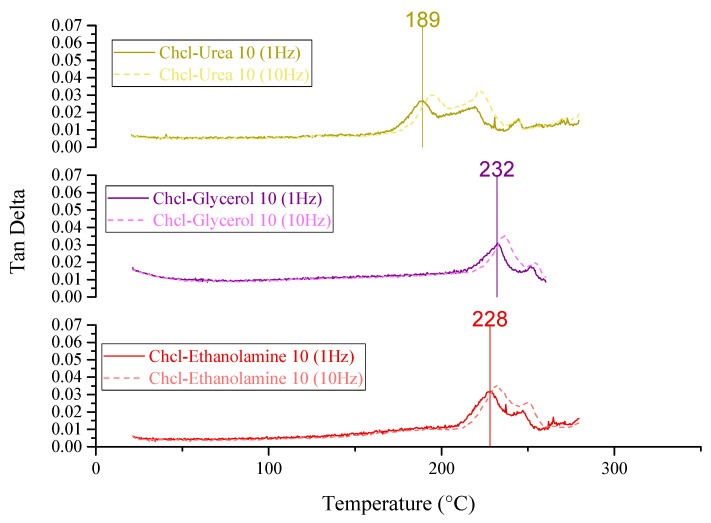
DMTA for PES/SAPO-34/DES using 10 *w*/*w*% at 1 Hz and 10 Hz.

**Figure A1:**
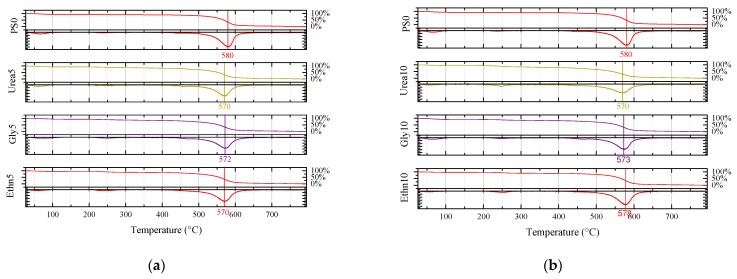
TGA for PES/SAPO-34 and PES/SAPO-34/DES membranes containing (**a**) 5 *w*/*w*% and (**b**) 10 *w*/*w*% of each DES.

PES/SAPO-34 membranes were prepared according to the methodology described in Section 3.2. DMTA and TGA were realized to compare thermal stability and flexibility based on the glass transition temperature for PES, PES/SAPO-34, and MMMs containing the 5 *w*/*w*% and 10 *w*/*w*% of DES, as depicted in Figure 1, Figure 2 and Figure 3 and Figure A1, respectively.

DMTA, in Figure 1, presents the glass transition temperature (T_g_) at a 1 Hz frequency of 234 °C and 219 °C for neat PES and PES/SAPO-34, respectively. Those temperatures express the flexibility degree of the membranes showing that the inclusion of SAPO-34 does not lead to a rigidified polymer/zeolite interface, since those rigidified interfaces would lead to a higher T_g_ than neat PES.

Figure 2 presents the glass transition temperature (Tg) at a 1 Hz frequency of 221 °C, 223 °C, and 198 °C for PES/SAPO-34/ChCl-urea, PES/SAPO-34/ChCl-glycerol, and PES/SAPO-34/ChCl-ethanolamine 5 *w*/*w*% concentrations. The T_g_ obtained for PES/SAPO-34/ChCl-urea and PES/SAPO-34/ChCl-glycerol are similar to that of the PES/SAPO-34 membrane. Moreover, the decrease in T_g_ for PES/SAPO-34/ChCl-ethanolamine shows that this concentration could lead to a more flexible membrane.

Figure 3 presents the glass transition temperature (Tg) at a 1 Hz frequency of 189 °C, 232 °C, and 228 °C for PES/SAPO-34/ChCl-urea, PES/SAPO-34/ChCl-glycerol, and PES/SAPO-34/ChCl-ethanolamine 10 *w*/*w*% concentrations. The decrease in T_g_ with an increased concentration of ChCl–urea obtained for PES/SAPO-34/ChCl-urea is related to higher flexibility and more polymer chain mobility. The increased concentration of ChCl–glycerol and ChCl–ethanolamine in MMMs promoted a rigidified membrane structure that could lead to less particle–polymer interaction.

Figure A1 presents the TGA for the membranes prepared in this work to compare the thermal stability of neat PES and PES/SAPO-34 mixed-matrix membranes with the modified membranes containing 5 and 10 *w*/*w*% of different DESs. The degradation temperature for PES/SAPO-34 is 581 °C. It seems that 5 *w*/*w*% and 10 *w*/*w*% of DES were not enough to cause an impact on the thermal stability of the membranes prepared. The DES degraded around 250 °C as presented in Figure 2 and Figure 3, which can also be observed in Figure A1 in the slight decrease in the same temperature scale.

**Figure A2:**
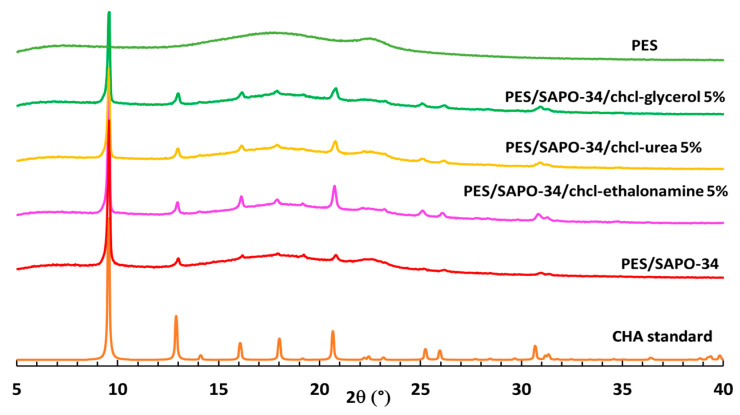
XRD for PES/SAPO-34 and PES/SAPO-34/DES membranes with 5 *w*/*w*% of DES.

Figure A2 presents the XRD for the membranes prepared with 5 *w*/*w*% of DESs compared to a neat PES membrane and a PES/SAPO-34 membrane without additives. CHA is the standard for characterizing SAPO-34 using XRD, endorsed by the International Zeolite Association (IZA). In CHA and SAPO-34, an identical tetrahedral structure is observed, with each tetrahedron coordinated by four oxygen atoms. These oxygen atoms serve as bridges between neighbouring tetrahedral atoms, resulting in a zeolite structure with uniform planes of dispersion. In Figure A2, the XRD obtained shows the neat PES pattern in all membranes and a CHA pattern of SAPO-34 with characteristic peaks in corresponding diffraction angles as the CHA standard diffractogram. The patterns of PES/SAPO-34 membranes with and without DES treatment are very similar. They are a combination of polymer and SAPO-34. The DES treatment did not influence the patterns.

**Figure A3:**
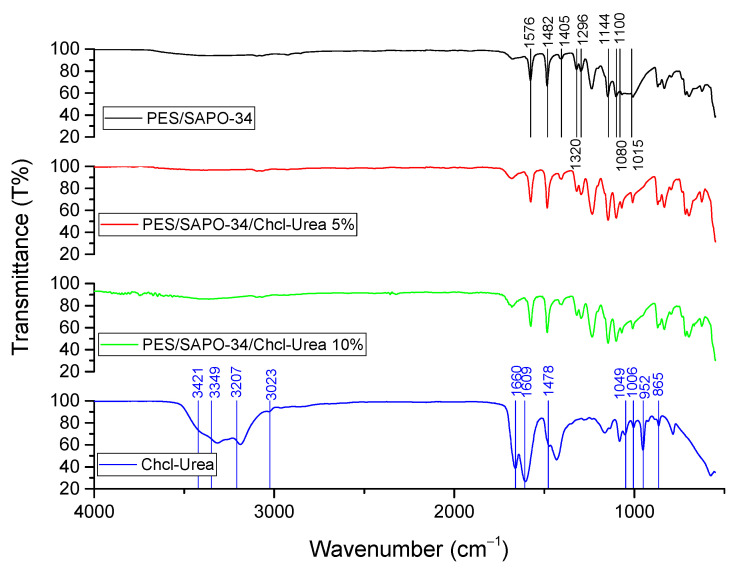
FTIR for PES/SAPO-34 and PES/SAPO-34/ChCl–urea membranes.

**Figure A4:**
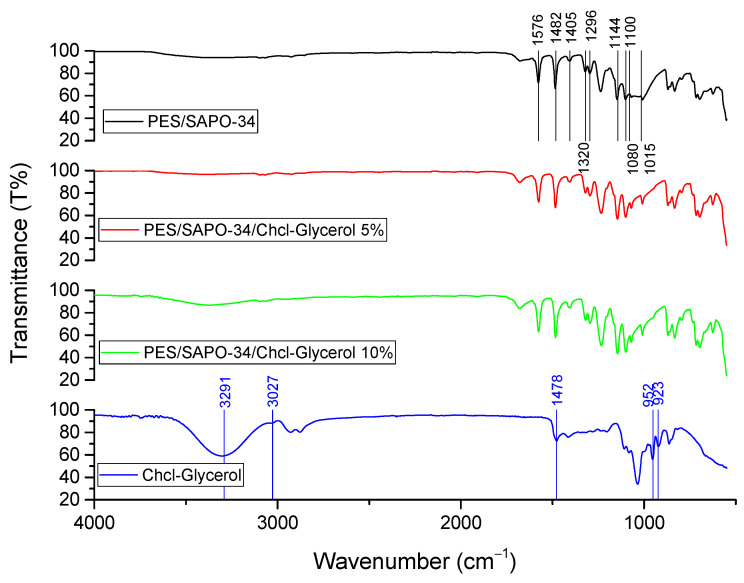
FTIR for PES/SAPO-34 and PES/SAPO-34/ChCl-glycerol membranes.

**Figure A5:**
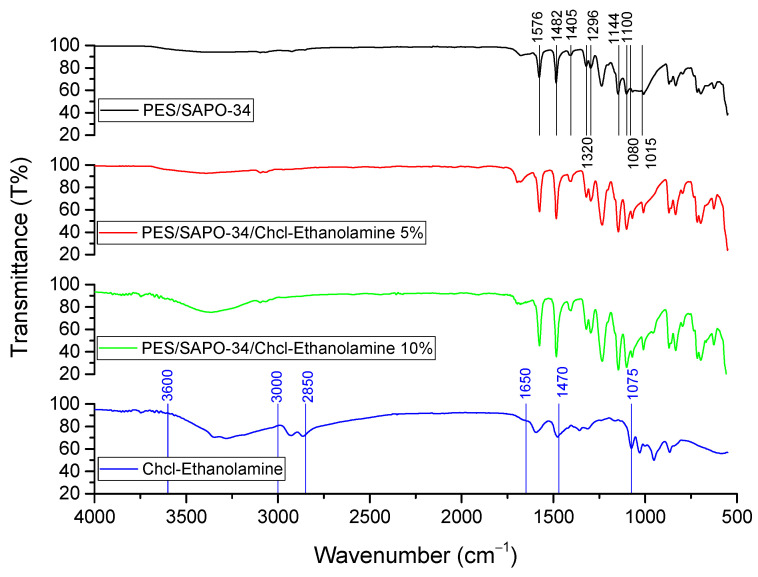
FTIR for PES/SAPO-34 and PES/SAPO-34/ChCl-ethalonamine membranes.

Moreover, Figure A3, Figure A4 and Figure A5 present the FTIR analysis for the membranes prepared and compared with the PES/SAPO-34 membrane and deep eutectic solvents related to those membranes. All membranes presented the three peaks observed at 1576–1577 cm^−1^, 1482–1484 cm^−1^, and 1405–1406 cm^−1^, indicating the presence of benzene rings. The two peaks at 1320–1321 cm^−1^ and 1296–1297 cm^−1^ indicate the presence of ether function. The two peaks at 1144–1146 cm^−1^ and 1100–1101 cm^−1^ indicate the presence of the sulfone group. All spectra are according to FTIR spectra for PES in the literature. The band between 1080 and 1015 cm^−1^ is related to Si-O and Al-O vibration and implies the addition of SAPO-34. The difference in the spectra’s shape between the PES/SAPO-34 and PES/SAPO-34/DES membranes in the range 1050–900 cm^−1^ denotes the existence of ChCl in the membrane since this alteration occurred in all membranes where ChCl was used and corresponds to the expected results since ChCl tends to degrade at 303 °C [36,37].

In Figure A3, no peaks characterizing urea could be found in the spectra for those membranes, suggesting the degradation of this compound after membrane drying [38].

In Figure A4, the peaks presented in 3291 cm^−1^, 3027 cm^−1^, 952 cm^−1^, and 923 cm^−1^ could be related to glycerol combined with ChCl, which is more present in the PES/SAPO-34/glycerol 10 *w*/*w*% membrane, suggesting the presence of this compound after membrane drying since glycerol degrades at 290 °C [39].

In Figure A5, the peak presented in 3600–3000 cm^−1^ is related to an OH band, and this could be related to ethanolamine presence. Although, ethanolamine’s degradation temperature is 167 °C, the OH bandwidth presented in the spectra is the product from that degradation [40].

**Figure 4:**
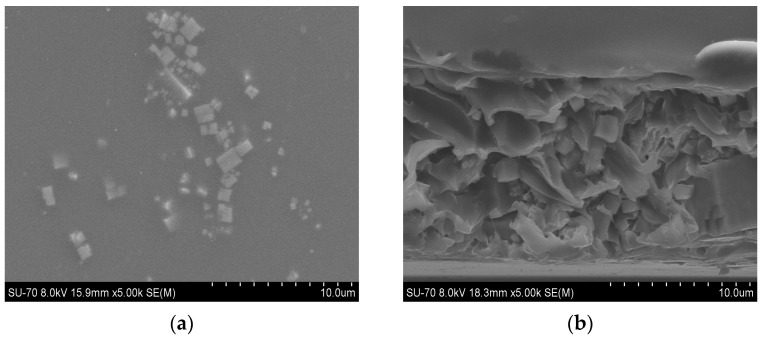
(**a**) Surface and (**b**) cross-section of PES/SAPO-34 membrane.

**Figure 5:**
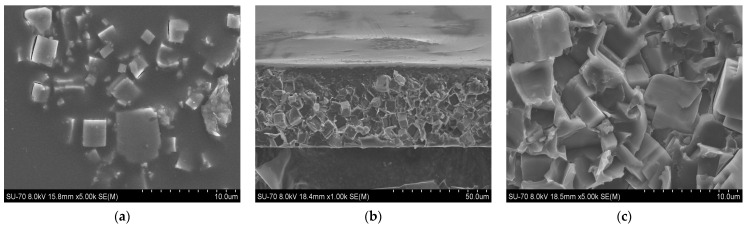
(**a**) Surface, (**b**) cross-section, and (**c**) zoom-in of particles in cross-section of PES/SAPO-34/ChCl-urea 5 *w*/*w*% membrane.

**Figure 6:**
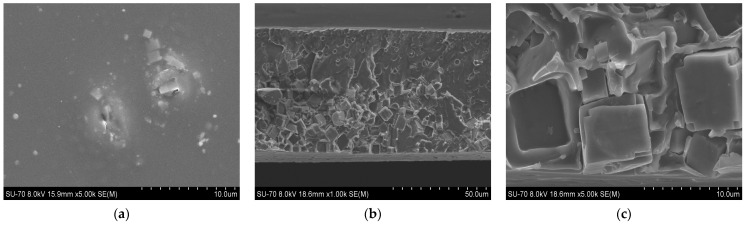
(**a**) Surface, (**b**) cross-section, and (**c**) zoom-in of particles in cross-section of PES/SAPO-34/ChCl-glycerol 5 *w*/*w*% membrane.

**Figure 7:**
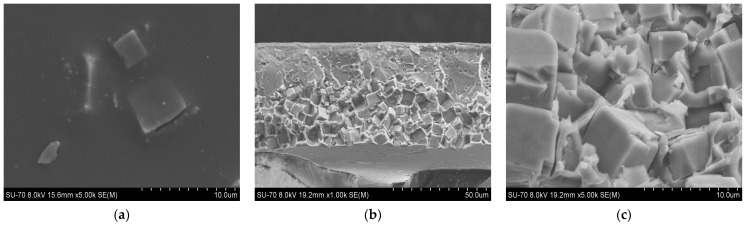
(**a**) Surface, (**b**) cross-section, and (**c**) zoom-in of particles in cross-section of PES/SAPO-34/ChCl-ethanolamine 5 *w*/*w*% membrane.

**Figure 8:**
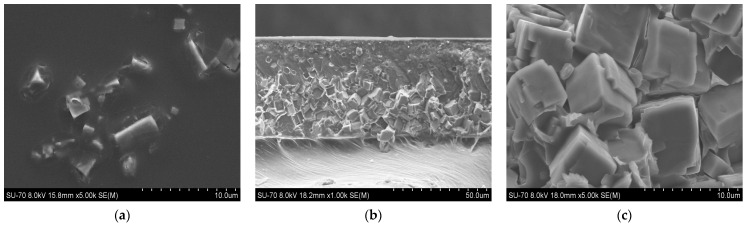
(**a**) Surface, (**b**) cross-section, and (**c**) zoom-in of particles in cross-section of PES/SAPO-34/ChCl-urea 10 *w*/*w*% membrane.

**Figure 9:**
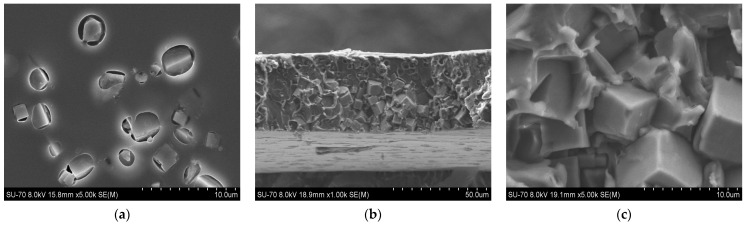
(**a**) Surface, (**b**) cross-section, and (**c**) zoom-in of particles in cross-section of PES/SAPO-34/ChCl-glycerol 10 *w*/*w*% membrane.

**Figure 10:**
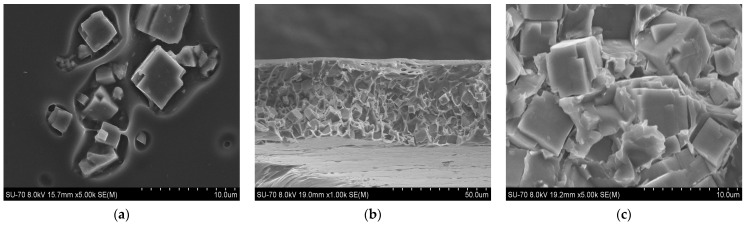
(**a**) Surface, (**b**) cross-section, and (**c**) zoom-in of particles in cross-section of PES/SAPO-34/ChCl-ethanolamine 10 *w*/*w*% membrane.

SEM analysis results for the PES/SAPO-34 and PES/SAPO-34/DES membranes containing 5 *w*/*w*% and 10 *w*/*w*% of the additives are presented in Figure 4, Figure 5, Figure 6, Figure 7, Figure 8, Figure 9 and Figure 10, respectively, depicting the membrane surface; cross-section of the general structure used to evaluate the crystal dispersion; and zoom-in of the particle–polymer interaction.

Figure 4a presents the PES/SAPO-34 membrane surface with crystals dispersed all over. Figure 4b presents the SEM analysis of the PES/SAPO-34 membrane cross-section, and the agglomeration of crystals with the polymeric matrix can be seen.

Figure 5, Figure 6, Figure 7, Figure 8, Figure 9 and Figure 10 present the SEM analysis of the PES/SAPO-34 membrane containing 5 and 10 *w*/*w*% of ChCl–urea, ChCl–glycerol, and ChCl–ethanolamine additives. Figure 5a, Figure 6a, Figure 7a, Figure 8a, Figure 9a and Figure 10a present a similar surface characterized by the presence of crystals.

In Figure 5b, Figure 6b, Figure 7b, Figure 8b, Figure 9b and Figure 10b, the cross-section of membranes with additives presented a higher concentration of crystals in the bottom phase. The addition of DES reduced the polymer viscosity, enabling the zeolite crystals to more easily precipitate inside the polymer matrix. Although this precipitation caused a higher concentration of particles on one side, forming an asymmetric membrane, it is worth pointing out that the particles were all covered by the polymer. This interaction seems stronger with the 5 *w*/*w*% DES concentration. Furthermore, an increase in the DES concentrations from 5 to 10 *w*/*w*% led to a rigidified polymer structure, as shown in Figure 3, consequently occluding the particles and reducing the particle–polymer interaction, which is more evident in Figure 5c, Figure 6c, Figure 7c, Figure 8c, Figure 9c and Figure 10c, which presents a zoom-in of the particles for each membrane. The interaction between the particles and polymer seems better in membranes containing 5 *w*/*w*% of DES in comparison to membranes with 10 *w*/*w*%.

### 2.2. Gas Permeation Tests

The films were submitted to a permeation test using CO_2_ and N_2_ pure gasses, and in selected membranes, CH_4_ was used. CO_2_ and N_2_ permeabilities and the ideal CO_2_/N_2_ selectivity were obtained using Equations (1) and (2); the average values are presented in Table 1.

In Table 1, a comparison between the neat PES and PES/SAPO-34 membranes reveals a reduction in the CO_2_ and N_2_ permeabilities. This reduction can be attributed to adding molecular sieving materials, which introduce an extended diffusion pathway. This extended pathway arises if the molecular sieve mechanism is the limiting process, as it compels molecules to traverse a longer distance compared to neat polymer membranes. The elongated path through the polymer (via the solution–diffusion mechanism) and the material’s pore structure (via the molecular sieve mechanism) results in prolonged permeation times, leading to lower permeability. However, despite this decrease in permeability, the properties of the molecular sieve material contribute to an increase in selectivity by more effectively retaining larger molecules [41,42].

**Figure 11:**
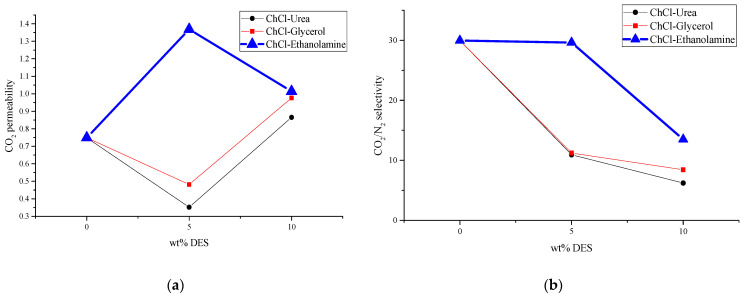
Comparison of the impact on (**a**) CO_2_ permeability and (**b**) CO_2_/N_2_ selectivity according to the amount of DES added.

The inclusion of choline chloride–urea and choline chloride–ethanolamine at a concentration of 5 *w*/*w*% resulted in a decrease in CO_2_ permeability. Conversely, choline chloride–glycerol at the same concentration led to an increase in CO_2_ permeability, as depicted in Figure 11a, while maintaining the CO_2_/N_2_ selectivity of the unmodified PES/SAPO-34 membrane, as shown in Figure 11b. Upon comparing the results obtained from gas permeation tests—Dynamic Mechanical Thermal Analysis (DMTA), Figure 2; FTIR, Figure A4; and scanning electron microscopy (SEM) analysis, Figure 5, Figure 6 and Figure 7—it can be inferred that the addition of 5 *w*/*w*% of deep eutectic solvent (DES) enhanced the interaction between particles and polymer, thereby preserving the original characteristics of the PES/SAPO-34 membrane.

Increasing the DES concentration to 10 *w*/*w*% increased CO_2_ permeability for choline chloride–urea and choline chloride–ethanolamine. In contrast, a reduction in CO_2_ permeability was observed when increasing the concentration of choline chloride–glycerol from 5 to 10 *w*/*w*%. In comparing the results obtained from gas permeation tests (DMTA in Figure 3, and SEM analysis in Figure 8, Figure 9 and Figure 10), it can be deduced that the addition of 10 *w*/*w*% of DES promoted a rigidified polymer structure at the particle–polymer interface, as indicated by the increase in the glass transition temperature (Tg) in Figure 3 and the overall structural changes in Figure 8, Figure 9 and Figure 10. This rigidified region may lead to diminished interaction between polymer and particles, ultimately resulting in increased CO_2_ permeability for all membranes and a reduction in CO_2_/N_2_ selectivity, as illustrated in Table 1 and Figure 11b.

**Figure 12:**
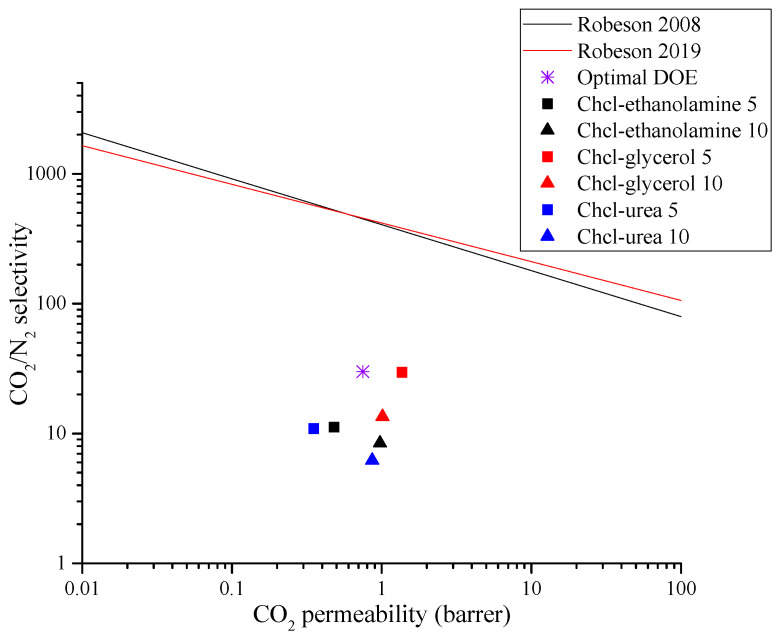
CO_2_/N_2_ Robeson upper bound [1,3] and a comparison of this work with SAPO-34 mixed-matrix membranes results.

**Figure 13:**
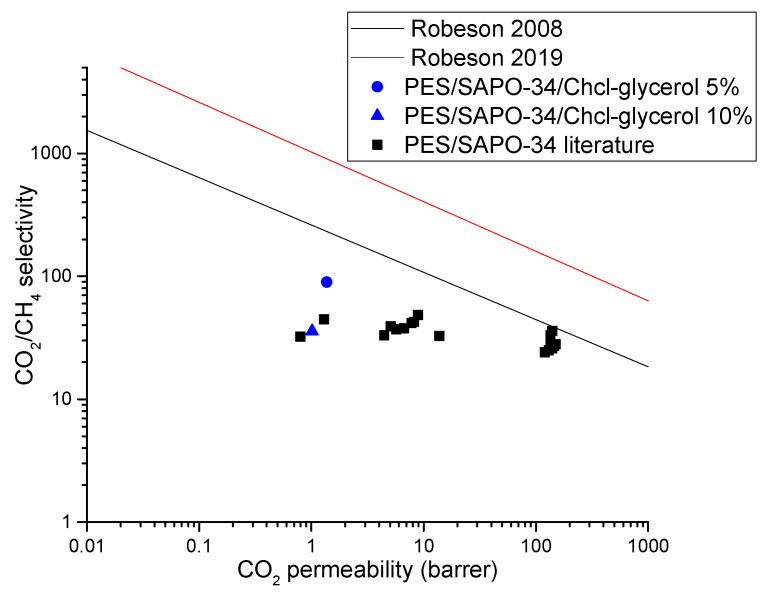
CO_2_/CH_4_ Robeson upper bound [1,3] and a comparison of this work with SAPO-34 mixed-matrix membranes results in the literature [42,43,44,45].

Among all PES/SAPO-34/DES membranes evaluated, the MMM containing 5 *w*/*w*% of ChCl–glycerol presented the best result, as shown in Figure 12, with double the CO_2_ permeability with a CO_2_/N_2_ selectivity similar to that of the non-modified PES/SAPO-34 membrane. The MMMs containing ChCl–glycerol were tested for CO_2_/CH_4_ separation. The results obtained are presented in Table 2 and Figure 13, showing that a high CO_2_ permeability was obtained with outstanding CO_2_/CH_4_ selectivity in PES/SAPO-34/ChCl-glycerol 5 *w*/*w*% when compared to other SAPO-34 MMMs in the literature.

The CO_2_/CH_4_ selectivity presented in Table 2 for PES/SAPO-34/ChCl-Glycerol 5 and 10 *w*/*w*% is on par with the expected results; CH_4_ having a larger kinetic diameter than N_2_ results in a higher separation performance of the pair CO_2_/CH_4_ compared to CO_2_/N_2_. PES/SAPO-34/ChCl-glycerol 5 *w*/*w*% showed outstanding CO_2_/CH_4_ selectivity with higher CO_2_ permeability when compared with the PES/SAPO-34 membrane. Figure 13 compares the ideal performance with other results in the literature.

## 3. Materials and Methods

### 3.1. Materials

The polymer used in this study was polyethersulfone (PES) from Goodfellow: transparent, 3 mm granular size, and molecular weight of 58,000 g/mol. It was used as the media for the membrane. The inorganic filler SAPO-34 was prepared using Aluminum isopropoxide (≥99% purity), phosphoric acid (85%), colloidal silica HS-40, morpholine (≥99% purity), and Tetraethylammonium hydroxide (TEAOH) solution (35 wt% in water) purchased from Sigma–Aldrich (St. Louis and Burlington, MA, USA) following the procedure used in a previous work [46]. PES and SAPO-34 were dried overnight at 105 °C and 200 °C, respectively, prior to use. The solvent used for membrane preparation and casting was N-methyl-pyrroline (NMP) (≥99.8% purity) from Roth. Choline chloride (ChCl) (≥98% purity), urea (≥99.5% purity), glycerol, and ethanolamine (99% purity), from Sigma-Aldrich, were used to prepare the respective deep eutectic solvents (DESs) following the molar composition shown in Table 3.

All DESs were selected due to their CO_2_ adsorption properties and prepared by weighing the corresponding amount for each molar ratio and mixing at 70 °C until a homogeneous solution was formed, and then cooled down [27,29,32,47].

### 3.2. Synthesis of PES/SAPO-34 Mixed-Matrix Membranes

PES/SAPO-34 membranes were prepared according to the mass composition of 15 wt% of PES and 18.5 wt% of SAPO-34, based on polymer mass in NMP; this was an optimal composition for the CO_2_/N_2_ separation developed in our previous work [48]. The SAPO-34 zeolite was added and stirred with N-Methyl-2-pyrrolidone (NMP) for 1 h and in an ultrasonic bath for 4 h. Then, a small quantity of PES was added to the NMP/SAPO-34 solution and mixed for 1 h at room temperature. The remaining PES was added and mixed until complete solubilization, and the solution was stirred overnight. The solutions were degassed for 1 h in ultrasonic bath and cast with a knife of 400 µm of initial thickness, dried at 90 °C for 8 h and at 200 °C for 20 h under vacuum. Membranes containing the deep eutectic solvents followed the same procedure. The DESs were previously prepared separately and the necessary amount, 5 or 10 *w*/*w*% of each DES, was added during the NMP/SAPO-34 mixing. The final thickness of the dried membranes was 50–60 µm.

### 3.3. Gas Permeation Experiments

Gas permeation experiments were conducted using pure gasses to determine their ideal permeability and diffusivity coefficients, and the results were compared to each other in a permeability unit called Barrer ($10^{-10}{\mathrm{cm}}_{STP}^{3}\cdot \mathrm{cm}/{\mathrm{cm}}^{2}\cdot s\cdot \mathrm{cmHg}$). The membranes were cut into 18 mm discs mounted on a steel permeation cell and tested at a feed pressure of 10 bar at room temperature. The permeabilities were obtained according to Equation (1).

(1) $$P_{i}=\frac{V_{s}l}{T_{AMB}\Delta p}\frac{T_{STP}}{p_{STP}A}\frac{dp}{dt}$$

The permeability of a certain gas (P_i_) is related to the thickness of the film (l), the fixed volume of the permeate (Vs), which is measured as standard. The ambient temperature is T_AMB_; the difference in pressure between the permeate and retentate, Δp; the permeation area, A; and the temperature and pressure in normal conditions, T_STP_ and P_STP_. The ideal selectivity results (α*_ab_*) are represented by the ratio between the permeability (*P*) of two pure gasses, a and b, respectively, measured separately under the same conditions in the same membrane to evaluate the separation performance, as presented in Equation (2).

(2) $$\alpha_{ab}=\frac{P_{a}}{P_{b}}$$

### 3.4. Characterization of PES/SAPO-34 Mixed-Matrix Membranes

The samples were characterized by a Dynamic Mechanical and Thermal Analysis (DMTA) using Triton Technology equipment, Leicestershire, UK, model Tritec 2000, with a heating rate of 2 °C/min in 1 and 10 Hz frequencies for all samples to verify the glass transition temperature, as well as a Thermogravimetric Analysis (TGA) using a TA Instruments TGA Q500 with an N2 flow of 10 mL/min at 10 °C/min to obtain the degradation temperatures. Fourier-transform infrared spectroscopy (FTIR) was used to compare the presence or absence of chemical components and alterations. A total attenuated reflectance non-destructive technique (ATR) was used in the range of 4000–550 cm^−1^ in a Perkin Elmer spectrometer model Spectrum Two FT-IR with a DTGS detector and a KBr beam-splitter, a resolution of 4.0 cm^−1^, 64 scans, and 50 N of constant applied force. Scanning electron microscopy (SEM) using a Hitachi SU-70 microscope was conducted to compare the membrane structure, morphology, and zeolite dispersibility, and a Contact Angle analysis was performed using Dataphysics equipment, a Contact Angle System OCA model, Filderstadt, Germany.

## 4. Conclusions

This work aimed to study the influence of choline chloride-based deep eutectic solvents containing urea, glycerol, or ethanolamine as additives into mixed-matrix membranes. Those DESs were added in situ during solution preparation in concentrations of 5 *w*/*w*% and 10 *w*/*w*%, based on polymer mass, in a fixed optimized composition of PES/SAPO-34 mixed-matrix membrane. FTIR analysis revealed the presence of glycerol in the membrane structures prepared. However, it also denoted the absence of urea and ethanolamine. DMTA showed that the addition of DES could lead to high membrane rigidification. While the addition of 5 *w*/*w*% of DES does not cause high disturbance in the polymer’s overall structure and flexibility, which helps with improving the accommodation of the crystal with a more flexible polymer, concentrations higher than 10 *w*/*w*% of DES disrupt the membrane stability, causing high rigidification processes and leading to less selective membranes. However, those additives did not prejudice the thermal stability of the membranes; as presented in the TGA results, they affect the membrane separation performance. DES concentrations of 20 *w*/*w*% or higher cause an increased porosity and a deformed membrane structure. Moreover, SEM analysis revealed an asymmetric membrane structure and reinforced the results in DMTA where DES-incorporated membranes led to rigidified structures and lower zeolite–polymer interaction with higher concentrations of DES. FTIR analysis clarified that DES with components with higher degradation temperature could resist the membrane drying procedure, resulting in more selective and permeable MMMs.

The gas permeation results showed that the PES/SAPO-34 membrane containing 5 *w*/*w*% of ChCl–glycerol presented increased CO_2_ permeability due to CO_2_ affinity and similar CO_2_/N_2_ selectivity when compared to a non-modified PES/SAPO-34 membrane. Further tests using pure CH_4_ also revealed high CO_2_/CH_4_ selectivity when compared to other SAPO-34 membranes presented in the literature, which ultimately resulted in a membrane with an outstanding CO_2_/CH_4_ ideal separation performance.

Furthermore, deep eutectic solvents can act as additives to improve membrane performance as a more eco-friendly and less expensive alternative. However, further studies are necessary to compare the use of the same membrane composition and different additives to evaluate the performance of particle surface modification.

## Figures and Tables

**Table 1:** Comparison of the CO_2_/N_2_ separation performance of PES, PES/SAPO-34, PES/SAPO-34/ChCl-urea, PES/SAPO-34/ChCl-glycerol, and PES/SAPO-34/ChCl-ethanolamine membranes.

Sample	*w*/*w*% DES	αCO_2_/N_2_	PCO_2_ (Barrer)	PN_2_ (Barrer)
Neat PES	0	21.35 ± 3.62	1.04 ± 0.003	0.05 ± 0.009
PES/SAPO-34	0	29.96 ± 0.67	0.75 ± 0.11	0.025 ± 0.0004
PES/SAPO-34/ChCl-urea	5	10.92 ± 2.03	0.35 ± 0.07	0.032 ± 0.0001
	10	6.21 ± 0.73	0.86 ± 0.07	0.139 ± 0.01
PES/SAPO-34/ChCl-glycerol	5	29.63 ± 2.57	1.37 ± 0.12	0.046 ± 0.00002
	10	13.49 ± 0.33	1.01 ± 0.04	0.075 ± 0.01
PES/SAPO-34/ChCl-ethanolamina	5	11.22 ± 0.29	0.48 ± 0.09	0.043 ± 0.01
	10	8.43 ± 0.46	0.98 ± 0.08	0.115 ± 0.003

**Table 2:** Comparison in CO_2_/CH_4_ separation performance of 5 and 10 *w*/*w*% PES/SAPO-34/ChCl-Glycerol membranes.

Sample	*w*/*w*% DES	αCO_2_/CH_4_	PCO_2_ (Barrer)	PCH_4_ (Barrer)
PES/SAPO-34/ChCl-Glycerol	5	89.70 ± 5.24	1.37 ± 0.12	0.02 ± 0.00043
	10	35.77 ± 0.62	1.01 ± 0.04	0.028 ± 0.001

**Table 3:** Deep eutectic solvent ratio.

Reagents	Molar Ratio	DES	T (K)	P (Bar)	xCO_2_
ChCl	1:2	1ChCl/2Urea	313–333	10.0–127.3	5.1–30.9
Urea					
ChCl	1:2	1ChCl/2Glycerol	303–343	1.9–63.5	0.6–39.9
Glycerol					
ChCl	1:6	1ChCl/6Ethanolamine	298	10.0	11.0
Ethanolamine					

## Data Availability

All data underlying the results are available as part of the article, and no additional source data are required.

## Appendix A

In this section, the results obtained by TGA (Figure A1), membrane XRD (Figure A2), and FTIR for all membranes prepared are represented in Figure A3, Figure A4 and Figure A5.
